# A computational model for gonadotropin releasing cells in the teleost fish medaka

**DOI:** 10.1371/journal.pcbi.1006662

**Published:** 2019-08-22

**Authors:** Geir Halnes, Simen Tennøe, Trude M. Haug, Gaute T. Einevoll, Finn-Arne Weltzien, Kjetil Hodne

**Affiliations:** 1 Faculty for Science and Technology, Norwegian University of Life Sciences, Ås, Norway; 2 Centre for Integrative Neuroplasticity, University of Oslo, Oslo, Norway; 3 Department of Informatics, University of Oslo, Oslo, Norway; 4 Institute of Oral Biology, University of Oslo, Oslo, Norway; 5 Department of Physics, University of Oslo, Oslo, Norway; 6 Department of Basic Sciences and Aquatic Medicine, Norwegian University of Life Sciences, Campus Adamstuen, Oslo, Norway; University of Edinburgh, UNITED KINGDOM

## Abstract

Pituitary endocrine cells fire action potentials (APs) to regulate their cytosolic Ca^2+^ concentration and hormone secretion rate. Depending on animal species, cell type, and biological conditions, pituitary APs are generated either by TTX-sensitive Na^+^ currents (*I*_*Na*_), high-voltage activated Ca^2+^ currents (*I*_*Ca*_), or by a combination of the two. Previous computational models of pituitary cells have mainly been based on data from rats, where *I*_*Na*_ is largely inactivated at the resting potential, and spontaneous APs are predominantly mediated by *I*_*Ca*_. Unlike in rats, spontaneous *I*_*Na*_-mediated APs are consistently seen in pituitary cells of several other animal species, including several species of fish. In the current work we develop a computational model of gonadotropin releasing cells in the teleost fish medaka (*Oryzias latipes*). The model stands out from previous modeling efforts by being (1) the first model of a pituitary cell in teleosts, (2) the first pituitary cell model that fires sponateous APs that are predominantly mediated by *I*_*Na*_, and (3) the first pituitary cell model where the kinetics of the depolarizing currents, *I*_*Na*_ and *I*_*Ca*_, are directly fitted to voltage-clamp data. We explore the firing properties of the model, and compare it to the properties of previous models that fire *I*_*Ca*_-based APs. We put a particular focus on how the big conductance K^+^ current (*I*_*BK*_) modulates the AP shape. Interestingly, we find that *I*_*BK*_ can prolong AP duration in models that fire *I*_*Ca*_-based APs, while it consistently shortens the duration of the predominantly *I*_*Na*_-mediated APs in the medaka gonadotroph model. Although the model is constrained to experimental data from gonadotroph cells in medaka, it may likely provide insights also into other pituitary cell types that fire *I*_*Na*_-mediated APs.

## Author summary

Excitable cells elicit electrical pulses called action potentials (APs), which are generated and shaped by a combination of ion channels in the cell membrane. Since one type of ion channels is permeable to Ca^2+^ ions, there is typically an influx of Ca^2+^ during an AP. Pituitary cells therefore use AP firing to regulate their cytosolic Ca^2+^ concentration, which in turn controls their hormone secretion rate. The amount of Ca^2+^ that enters during an AP depends strongly on how long it lasts, and it is therefore important to understand the mechanisms that control this. Pituitary APs are generally mediated by a combination of Ca^2+^ channels and Na^+^ channels, and the relative contributions of from the two vary between cell types, animal species and biological conditions. Previous computer models have predominantly been adapted to data from pituitary cells which tend to fire Ca^2+^-based APs. Here we develop a new model, adapted to data from pituitary cells in the fish medaka, which APs that are predominantly Na^+^-based, and compare its dynamical properties to the previous models that fire Ca^2+^-based APs.

## Introduction

Several types of excitable cells elicit electrical pulses called action potentials (APs), which, depending on cell type, can trigger neurotransmitter release, cellular contraction, hormone release or other actions. APs are generated by a combination of ion channels in the plasma membrane, which are typically characterized by the type of ions they are permeable to, and their voltage and/or Ca^2+^ dependent gating properties. The primary role of APs in endocrine pituitary cells is to regulate the cytosolic Ca^2+^ concentration, which in turn controls the hormone secretion rate in these cells [1]. Hormone secretion often occurs as a response to input from the hypothalamus, peripheral endocrine glands, or from other pituitary cells. However, many endocrine cells are also spontaneously active [1–10]. The spontaneous activity is partly a means to regulate the re-filling of intracellular Ca^2+^ stores, but in several cells also leads to a basal release of hormones. An understanding of the mechanisms regulating the electrodynamics of these cells is therefore fundamental for understanding their overall functioning.

While neuronal APs are predominantly mediated by TTX-sensitive Na^+^ currents (*I*_*Na*_), AP generation in endocrine cells depends strongly on high-voltage-activated Ca^2+^ currents (*I*_*Ca*_), which in addition to their role in affecting the voltage dynamics of the cell, also are the main source of Ca^2+^ entry through the plasma membrane [3, 11, 12]. In some studies of endocrine cells, APs were exclusively mediated by *I*_*Ca*_, and the spontaneous membrane excitability was insensitive or nearly so to TTX [1, 2, 13–16]. In other studies, APs were evoked by a combination of *I*_*Ca*_ and *I*_*Na*_ [4, 7, 17–19]. In one of these studies, TTX was found to block single, brief action potentials, while action potentials of long duration and low amplitude persisted [18], indicating the roles and different time courses of the *I*_*Ca*_ and *I*_*Na*_ components. The strong involvement of *I*_*Ca*_ could explain why pituitary APs typically last longer (typically some tens of milliseconds [8]) than neuronal APs (a few milliseconds), which are mainly mediated by *I*_*Na*_.

All endocrine cells express *I*_*Na*_ [8], and TTX sensitive APs can typically be triggered by current injections from hyperpolarized holding potentials even in cells where they are not elicited spontaneously [4, 17, 20, 21]. The reason why the spontaneous activity in many cases is TTX insensitive is likely that a major fraction of *I*_*Na*_ is inactivated at the resting membrane potential [15, 16]. The reason why this is not always the case, may be that the resting potentials vary greatly between different studies. Only for rat somatotrophs, resting potentials ranging as wide as from −30 mV [13] to −80 mV [18] have been reported.

Computational models constructed to capture the essential activity of pituitary cells have predominantly relied on rat data. The typical resting potentials for rat pituitary cells lie in the range between −50 mV and −60 mV, and at these resting levels, *I*_*Na*_ tends to be inactivated and the spontaneous activity TTX insensitive (see reviews in [8, 22]). Models based on rat data have therefore typically excluded *I*_*Na*_ [3, 9, 23–30]. As TTX-sensitive spontaneous APs have been seen in mice corticotrophs [19], *I*_*Na*_ was included in a recent computational model of these cells [31], and in a more generic murine pituitary cell model based on the previous rat and mice models [32]. However, the role of *I*_*Na*_ in these models was mainly in modulating the firing patterns, and it was not essential for AP firing as such [31, 32]. Furthermore, *I*_*Ca*_ and *I*_*Na*_ were in these models described by simplified kinetics schemes that were adjusted to give the models the desired firing properties, but not explicitly adapted to voltage-clamp recordings of the respective currents in the cell species being modeled.

There are reasons to believe that the dynamical properties of the above cited pituitary cell models are not well suited to represent teleost pituitary cells. Firstly, TTX-sensitive spontaneous activity has been seen in goldfish gonadotrophs resting at −60 mV [4], and TTX sensitive APs has been evoked from a holding potential as high as −50 mV in pituitary cells in cod [7], suggesting that *I*_*Na*_ may be more available in resting pituitary cells in fish [4]. Secondly, data from gonadotrophs and somatotrophs in goldfish [4, 20] and unspecified pituitary cells in tilapia [5] show APs with very short duration (< 10 ms) compared to the APs in the previous rat models (several tens of ms), putatively indicative of a stronger involvement of *I*_*Na*_. A third difference between fish and rat pituitary cells may be in the role of the big conductance K^+^ current (*I*_*BK*_), which has been shown to have a paradoxical role in some rat pituitary cells, i.e., it can prolong the duration of *I*_*Ca*_-mediated APs, and sometimes give rise to pseudo-plateau bursts, contrary to what one would expect from a hyperpolarizing current [9, 25]. *I*_*BK*_ is almost absent in rat gonadotrophs [25], and this was proposed as an explanation to why these cells tend to be less bursty than other pituitary cell types in rats [1, 9, 32]. In contrast, *I*_*BK*_ is highly expressed in medaka gonadotrophs, but without making these cells bursty [12]. The indication that there are differences between rat and fish pituitary cells are further supported by experiments presented in the current work, performed on luteinizing hormone (LH)-producing gonadotroph cells in medaka. We show that these cells elicit brief spontaneous APs that, unlike spontaneous APs in the previous murine pituitary cell models, predominantly are mediated by TTX sensitive Na^+^ currents (*I*_*Na*_). Furthermore, we show that *I*_*BK*_ acts to make APs narrower in medaka gonadotrophs, and thus have the opposite effect from what they have in rat gonadotrophs.

As previous computational models based on murine data seem unsuited to describe the spontaneous activity of teleost pituitary cells, we here present a novel pituitary cell model constrained to data from medaka gonadotrophs. Given the putatively complex interplay between *I*_*Ca*_ and *I*_*Na*_ during the AP upstroke, we put extra effort into developing accurate models of these two currents, and constrained their kinetics to voltage-clamp recordings of the individual currents. In addition to *I*_*Ca*_ and *I*_*Na*_, we included a leak current and three K^+^ currents in the model. These we adopted from previous pituitary cell models, and adjusted to adapt the firing properties of the model to current-clamp recordings from medaka gonadotrophs under control conditions, after application of TTX, and after application of the *I*_*BK*_ blocker paxilline.

By comparing the medaka gonadotroph model, which predominantly fires *I*_*Na*_-mediated APs, with three previous models of murine pituitary cells, which predominantly [32] or exclusively [9, 27] fire *I*_*Ca*_-mediated APs, we explore the consequences of having different AP-generating mechanisms. We find that the medaka gonadotroph model produces spontaneous APs that are faster than those in the murine models, and thus more suited to describe the firing properties of teleost pituitary cells. Furthermore, we show that while *I*_*BK*_ may broaden APs in the murine pituitary models [9, 27, 32], it consistently had a narrowing effect on APs in the medaka gonadotroph model, and propose explanations to these model differences. By this, we add to the discussion of the role played by *I*_*BK*_ in shaping pituitary APs [8], and suggest that the effect of *I*_*BK*_ on APs is mainly determined by the timing of *I*_*BK*_-activation relative to the AP peak, as also proposed previously [9].

Although the model presented here was tailored to represent gonadotroph cells in medaka, we believe that it is of a more general value for improving our understanding of *I*_*Na*_-based APs in the pituitary, which are elicited by several endocrine cell types and in several animal species, depending on biological conditions [4, 7, 17, 18, 33–35].

## Results

### Characteristic response patterns of medaka gonadotrophs

**Fig 1:**
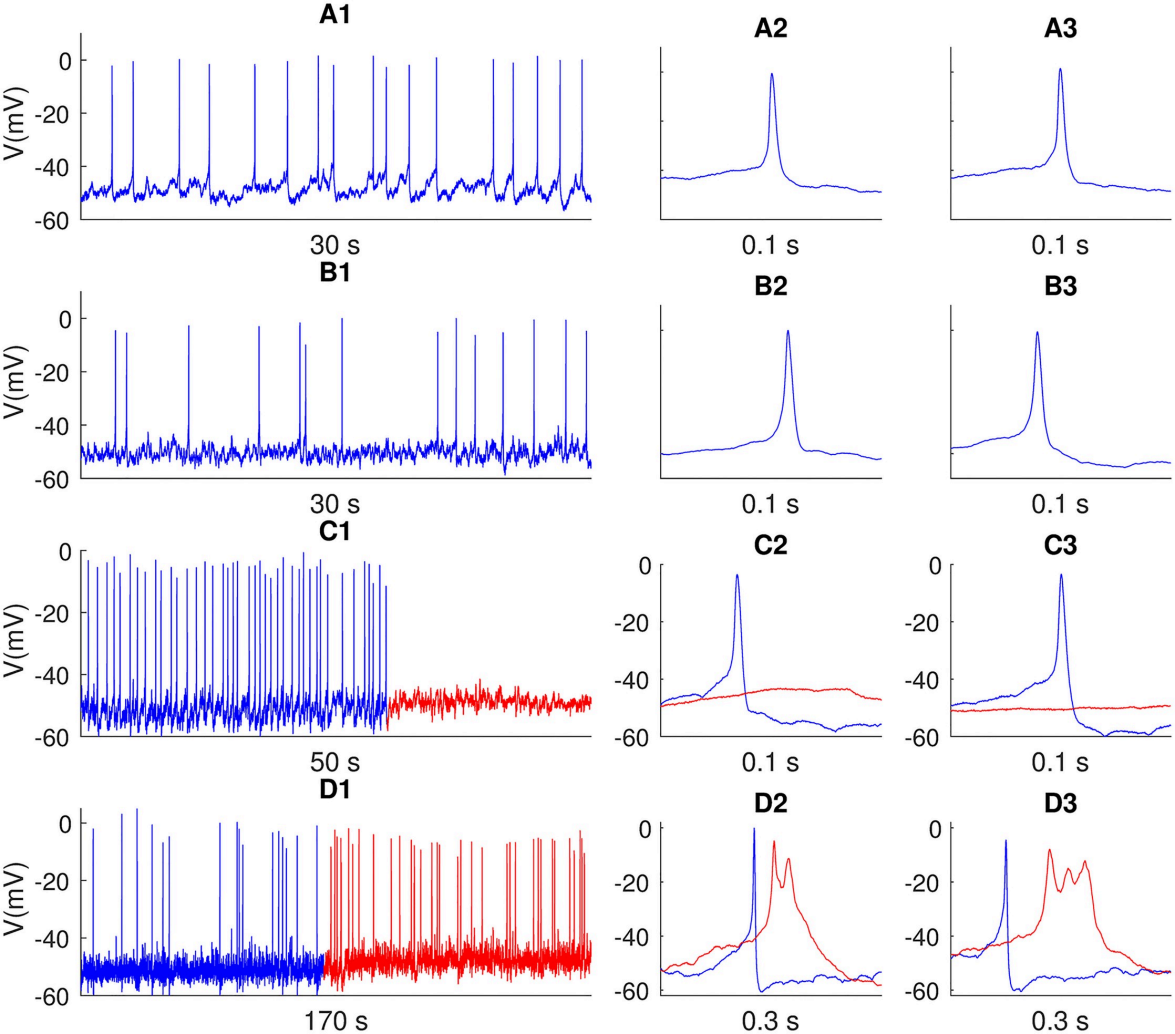
Experimental voltage recordings. (A1-B1) Spontaneous AP firing in two selected cells. (A2-A3) Close-ups of selected APs from the cell in A1. (B2-B3) Close-ups of selected APs from the cell in A2. (C1) Spontaneous activity before (blue) and after (red) TTX application. (C2-C3) Close-ups of two selected events before (blue) and after (red) TTX application. (D1) Spontaneous activity before (blue) and after (red) paxilline application. (D2-D3) Close-ups of two selected events before (blue) and after (red) paxilline application. The firing rates in the various recordings were 0.64 Hz (A1), 0.57 Hz (B1), 1.22 Hz (C1, before TTX), 0.17 Hz (D1, before paxilline) and about 0.35 Hz (D1, after paxilline). AP width (defined as the time between the upstroke and downstroke crossings of the voltage midways between −50 mV and the peak potential) varied between 3 and 7 ms, with mean width of 3.7 ms (A1), 4.9 ms (B1), 3.7 ms (C1, before TTX). In (D1), average AP widths were 4.2 ms before paxilline. After paxilline, the AP shapes varied, with AP widths ranging from 9 ms to 90 ms, with a mean of 25 ms. AP peak voltages varied between -11.8 mV and +5.5 mV, with mean peak values of −0.4 mV (A1), −3.4 mV (B1), −5.1 mV (C1, before TTX), −3.1 mV (D1, before paxilline), and −6.4 mV (D1, after paxilline). AP width was calculated at half max amplitude between −50 mV and AP peak. The experiments were performed on gonadotroph LH-producing cells in medaka. All depicted traces were corrected with a liquid junction potential of −9 mV. The time indicated below each panel refers to the duration of the entire trace shown.

The general electrophysiological properties of gonadotroph cells in medaka were assessed through a series of voltage-clamp and current-clamp experiments. The voltage-clamp experiments used to develop kinetics models of Na^+^ and Ca^2+^ currents are presented in the Methods section. Here, we focus on the key properties of spontaneous APs as recorded in current clamp. Selected, representative experiments are shown in Fig 1.

Although variations were observed, the medaka gonadotrophs typically had a resting potential around −50 mV, which is within the range found previously for goldfish [4] and cod [10] gonadotrophs. As for goldfish gonadotrophs, the majority of medaka gonadotrophs fired spontaneous APs with peak voltages slightly below 0 mV. The spontaneous APs were always regular spikes (i.e., not bursts or plateau-like) and the AP width varied between 3 and 7 ms (blue traces in Fig 1). Similar brief AP waveforms have been seen in previous studies on fish [4, 5, 20], while the APs reported for rat gonadotrophs are typically slower, i.e. from 10-100 ms [8]. The spontaneous AP activity was completely abolished by TTX application (Fig 1B).

Finally, we explored how paxilline (an *I*_*BK*_ blocker) affected the spontaneous activity of medaka gonadotrophs. In the experiment shown in Fig 1D, paxilline increased the firing rate and slightly reduced the mean AP peak amplitude, but these effects were not seen consistently in experiments using paxilline application. However, in all experiments, paxilline application was followed by a small increase of the resting membrane potential (Fig 1C), and a broadening of the AP waveform (Fig 1D2 and 1D3). Similar effects have been seen in goldfish somatotrophs, where application of tetraethylammonium (a general blocker of K^+^ currents) lead to broadening of APs [20]. The effect of *I*_*BK*_ in goldfish and medaka gonadotrophs is thus to make APs narrower, which is the opposite of what was found in rat pituitary somatotrophs and lactotrophs, where *I*_*BK*_ lead to broader APs and sometimes to burst-like activity [9, 25].

### A computational model of medaka gonadotrophs

The model for medaka gonadotrophs is described in detail in the Methods section, but a brief overview is given here. The dynamics of the membrane potential is determined by the differential equation:

$$\begin{array}{c} C_{m}\frac{dV}{dt}=-\left(I_{Ca}+I_{Na}+I_{K}+I_{BK}+I_{SK}+I_{leak}\right). \end{array}$$ (1)

The three K^+^ currents, *I*_*K*_, *I*_*BK*_ and *I*_*SK*_, were based on previously published models ([9, 32]), but adjusted (see Methods) so that the final model had an AP shape and AP firing rate that were in better agreement with the experimental data in Fig 1. *I*_*K*_ denotes the delayed rectifier K^+^ channel [9], *I*_*SK*_ denotes the small-conductance K^+^ channel, activated by the intracellular Ca^2+^ concentration [9], and *I*_*BK*_ denotes the big-conductance K^+^ channel. The latter was assumed to be both voltage and Ca^2+^-dependent. As it is often co-localized high-voltage-activated Ca^2+^ channels, it was assumed to sense a domain-Ca^2+^ concentration proportional to *I*_*Ca*_ [32].

**Fig 2:**
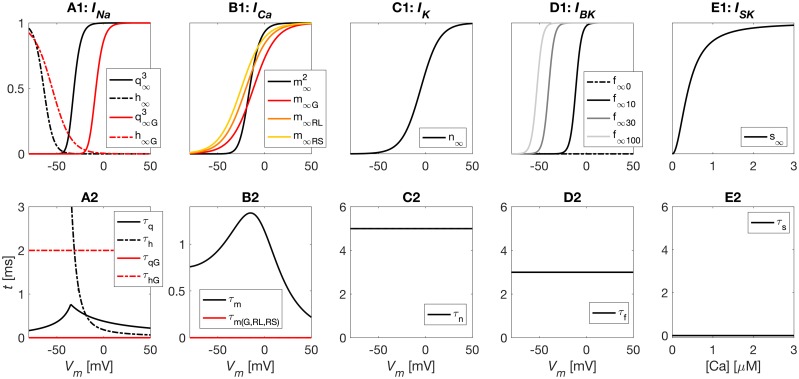
Ion channel kinetics. (A1) *I*_*Na*_ in models of a medaka gonadotroph and a generic murine pituitary cell (G). *I*_*Na*_ had three activation gates (*q*^3^) and one inactivation gate (*h*). (B1) *I*_*Ca*_ activation in models of medaka gonadotrophs, generic murine pituitary cells (G), rat lactotrophs (RL), and rat somatotrophs (RS). Two activation gates were used in medaka (*m*^2^), and one in the other models. (C1) *I*_*K*_ had one activation gate (*n*). (D1) *I*_*BK*_, had one activation gate (*f*), depending on voltage and domain [Ca^2+^], the latter assumed to be proportional to *I*_*Ca*_. Results shown for *I*_*Ca*_ = 0, 10, 30 and 100 pA. (E1) *I*_*SK*_ was Ca^2+^ activated with one activation gate *s*. (A2-B2) Voltage-dependent activation time constants were computed for *I*_*Na*_ (A2) and *I*_*Ca*_ (black curves), while all murine model used fixed time constants (red curves). Voltage-independent activation-time constants were used for *I*_*K*_ (C2), *I*_*BK*_ (D2) and *I*_*SK*_ (E2). (A-E).

The depolarizing membrane currents consisted of a high-voltage activated Ca^2+^ current (*I*_*Ca*_) and a Na^+^ current (*I*_*Na*_), both of which were novel for this model, and adapted to new voltage-clamp data from gonadotroph cells in medaka (see Methods). *I*_*Na*_ activated in the range between −50 mV and −10 mV, with half activation at −32 mV (black curves, Fig 2A1), quite similar to what was previously found in goldfish gonadotrophs [4]. *I*_*Na*_ inactivated in the range between −90 mV and −40 mV, with half-inactivation at −64 mV, which was lower than in goldfish, where the half-inactivation was found to be around −50 mV [4]. With the activation kinetics adapted to medaka data, only 6% of *I*_*Na*_ was available at the typical resting potential of −50 mV. The fact that medaka still showed TTX-sensitive spontaneous activity thus suggests that *I*_*Na*_ is highly expressed in these cells. In comparison, in the generic murine pituitary model [32], *I*_*Na*_ activation required depolarization to voltages far above the resting potential (red curves, Fig 2A1)), meaning that this model could not elicit spontaneous *I*_*Na*_-based APs.

Both *I*_*Na*_ and *I*_*Ca*_ had fast activation in medaka gonadotrophs, *I*_*Na*_ being slightly faster with a time constant of about 0.5–0.8 ms in the critical voltage range (Fig 2A2), whereas *I*_*Ca*_ had a time constant >1 ms in the critical voltage range (Fig 2B2). *I*_*Ca*_ activated in the range between −40 mV and +10 mV, with a half activation at 16 mV (red curve in Fig 2B1). This activation curve was much steeper than *I*_*Ca*_ in the rat models (colored curves). The high activation in medaka gonadotrophs threshold suggests that *I*_*Ca*_ is unsuitable for initiating spontaneous APs, making spontaneous activity critically dependent on *I*_*Na*_, unlike in all rat models, where *I*_*Ca*_ was highly available around the resting potential.

### BK currents cause briefer APs in medaka gonadotrophs

**Fig 3:**
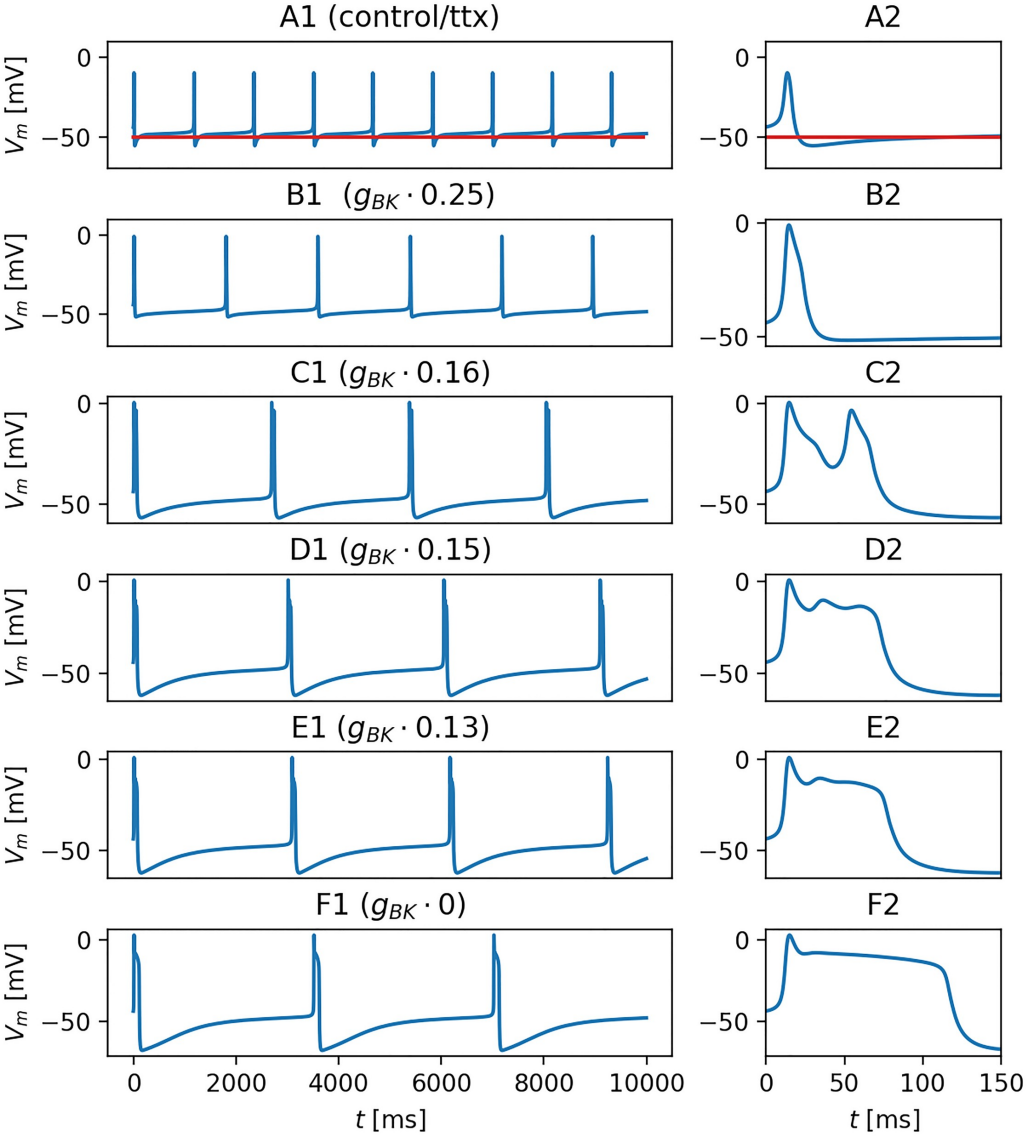
Effects of *I*_*BK*_ on AP shape. The spontaneous activity of the medaka gonadotroph model for different levels of BK-expression. (A1) Spontaneous activity under control conditions, where *g*_*BK*_ was maximally expressed (blue curve, *g*_*BK*_ = 0.31mS/cm^2^). AP firing was completely abolished by setting *g*_*Na*_ = 0, mimicking the effect of TTX (red curve). (B1-F1) Simulations with *g*_*BK*_ reduced to fractions (indicated above panels) of the maximum value. Reductions in *g*_*BK*_ consistently lead to broader APs. (A2-F2) Close-ups of the first APs in seen in A1-F1.

With the right tuning, the medaka gonadotroph model reproduced the essential firing patterns seen in the experiments (Fig 1). In control conditions, it fired sharp APs (AP width was 6 ms) with relatively low peak voltages (around -10 mV), and had a spontaneous firing rate slightly below 1 Hz (Fig 3A). AP firing was completely abolished when the Na^+^-conductance *g*_*Na*_ was set to zero, mimicking the effect of TTX application in the experiments.

A series of previous models have shown that *I*_*BK*_ may act to broaden APs and promote bursting in rat pituitary cells [9, 25, 27, 32]. In contrast, a high *I*_*BK*_ expression in medaka gonadotrophs [12] does not make these cells bursty. On the contrary, the experiments in Fig 1D showed that medaka gonadotrophs fired broader APs when BK channels were blocked. This was also seen in the model simulations, when the BK-channel conductance (*g*_*BK*_) was reduced relative to its value during control (Fig 3B–3F). Generally, a reduction in *g*_*BK*_ lead to a broadening of the AP event. The resemblance with paxilline data was strongest in simulations with partial reduction, such as in Fig 3C and 3D, where *g*_*BK*_ had been reduced to 16% and 15% of its control value, respectively. Then, AP events were about 60 ms wide, and included plateau potentials that presumably reflected an interplay between *I*_*Ca*_ and repolarizing currents activating/inactivating after the initial AP peak. When *g*_*BK*_ was set to zero, the oscillations were not seen, and the AP was prolonged by a flatter and more enduring > 100 ms plateau. It is reasonable to assume that also in the experiments, the blockage of BK by paxilline was not complete.

### Membrane mechanisms controlling the AP width

**Fig 4:**
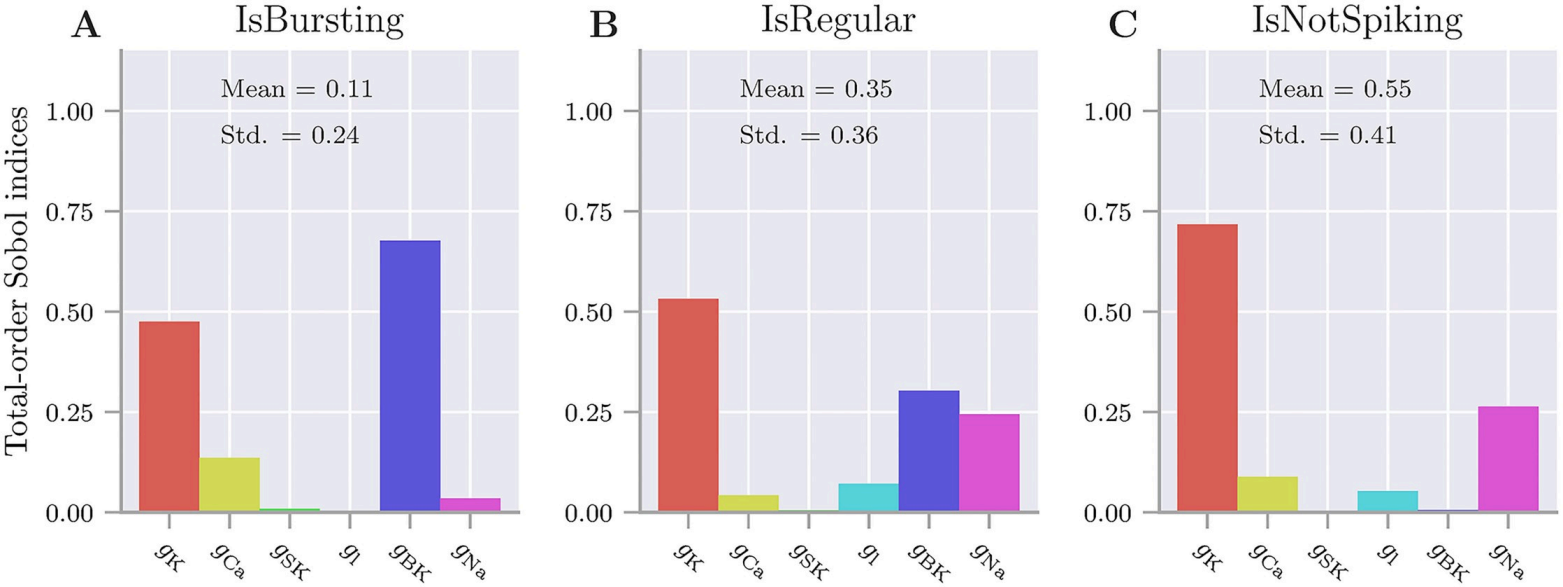
Feature-based sensitivity analysis. Sensitivity to variations in the maximum ion-channel conductances. The analysis summarizes a large number of simulations where the maximum conductances of all ion channels were varied within intervals ±50% of their original values. An exception was made for *g*_*BK*_, which was varied between 0 and 0.31 mS/cm^2^. Features were binary (0 or 1), and (A) *IsBursting* = 1 for simulations that elicited one or more bursts, (B) *IsRegular* = 1 for simulations that elicited one or more regular APs, and (C) *IsNotSpiking* = 1 for simulations that did not elicit any AP events. (A-C) Histograms depict the total-order Sobol sensitivity indices which quantify how much of the variability in a response features that is explained by the variation of a given model parameter, including all its co-variances with other parameters. The analysis was performed by aid of the recently developed toolbox *Uncertainpy* [36] (see Methods for details).

To explore in further detail how the various membrane mechanisms affected the AP firing, we performed a feature-based sensitivity analysis of the medaka gonadotroph model (Fig 4). We then assigned the maximum conductances of all included currents uniform distributions within intervals ±50% of their default values (Table 1), and quantified the effect that this parameter variability had on selected aspects of the model output (see Methods). An exception was made for *g*_*BK*_, which was assigned a uniform distribution between 0 and the maximum value given in Table 1), i.e., from fully available to fully blocked, motivated by the fact that this was the possible range spanned in the paxilline experiments (Fig 1D). We note the total-order Sobol sensitivity indices considered in the current analysis reflects complex interactions between several nonlinear mechanisms, and that mechanistic interpretations therefore are difficult. Below, we have still attempted to extract the main picture that emerged from the analysis.

**Table 1:** Conductances in the default parameterization of the medaka gonadotroph model. *g*_*BK*_ was varied between simulations, and had values between 0 and the (maximum) value given the table. * *g*_*Ca*_ had the units of a permeability.

parameter	value	units
*g*_*Na*_	21.9	mS/cm^2^
*g*_*Ca*_	0.06	cm/s*
*g*_*K*_	0.42	mS/cm^2^
*g*_*BK*_	0.31	mS/cm^2^
*g*_*SK*_	0.40	mS/cm^2^
*g*_*leak*_	0.02	mS/cm^2^

Three features of the model responses were considered: (i) *IsBursting*, (ii) *IsRegular*, and (iii) *IsNotSpiking*. Following the definition used by Tabak et al. [9], plateau potentials of duration longer than 60 ms (such as those in Fig 3C2–3F2) were defined as bursts. For simplicity, we used the definition loosely, and referred to enduring plateau potentials as bursts even in cases where they did not contain any oscillations (such as in Fig 3C2–3F2)). APs of shorter duration than this (such as those in Fig 3A2 and 3B2) were defined as regular spikes. All the features (i-iii) were binary, meaning e.g., that *IsBursting* was equal to 1 in a given simulation if it contained one or more bursts, and equal to 0 if not. The mean value of a feature (taken over all simulations) then represented the fraction of simulations that possessed this feature. For example, *IsBursting* had a mean value of 0.11, *IsRegular* had a mean value of 0.35, and *IsNotSpiking* had a mean value of 0.55, which means, respectively, that 11% of the model parameterizations fired bursts, 35% fired regular APs, and 55% did not fire any kind of APs. AP activity was thus seen in less than half of the parameterizations. This reflects that the default configuration had a resting potential only slightly above the AP generation threshold, so that any parameter re-sampling that would make the cell slightly less excitable, would abolish its ability for AP generation. We note that the mean values of the three features sum up to 1.01 and not to unity. This was because a few of the parameterizations fired both bursts and regular APs within the same simulation.

The total-order Sobol indices, shown in histograms in Fig 4, quantify how much of the variability (between different simulations) in the response features that are explained by the variation of the different model parameters, i.e., the maximum conductances. When interpreting these results, we should keep in mind that the feature sensitivities are not independent, i.e., if *Isbursting* equals 0 for a given implementation, it means that either *IsRegular* or *IsNotSpiking* must equal 1. When the sensitivity to *g*_*Na*_ was small for *IsBursting*, but quite large for *IsRegular* and *IsNotspiking*, it then means that *g*_*Na*_ was important for switching the model between not firing and regular firing, while it contributed less to prolonging the APs into possible bursts. In contrast,*IsNotSpiking* was almost insensitive to *g*_*BK*_, while *IsBursting* and *IsRegular* had a high sensitivity to *g*_*BK*_. A little simplified, we can thus say that *g*_*Na*_ determined whether the model fired an AP (Fig 4C), while *g*_*BK*_ determined whether the AP, if fired, became a burst or a regular spike (Fig 4A and 4B).

All three features had a high sensitivity to *g*_*K*_, which indicates that *g*_*K*_ played multiple roles for the firing properties of the model. Firstly, *I*_*K*_ had a nonzero activity level around rest (cf. Fig 2C1), and was important (along with *I*_*Na*_ and the leakage current, *I*_*l*_) for determining whether the resting potential was above the AP threshold, hence the high sensitivity of *IsNotSpiking* to *g*_*K*_. Having a broad activation range, *I*_*K*_ was also important for repolarizing the membrane potential after the AP peak, and thus for the duration of the AP. Therefore, also *IsBursting* had a high sensitivity to *g*_*K*_.

The mechanisms behind burst generation are reflected in the sensitivity of *IsBursting* to *g*_*BK*_, *g*_*K*_ and *g*_*Ca*_. Here, *I*_*Ca*_ is responsible for mediating the plateaus that prolong APs into possible bursts, while *g*_*BK*_ and *g*_*K*_ may prevent bursts by facilitating a faster down-stroke. The interaction between *g*_*BK*_ and *g*_*K*_ in mediating the AP downstroke is complex, as we comment on further in the next subsection. The sensitivity to the last K^+^-channel, *g*_*SK*_, was very low in all the features considered here. *g*_*SK*_ had very little impact on the AP shape or the ability of the model to elicit APs, but was included in the model since it was important for regulating the firing rate.

### BK currents have opposite effects on AP shape in different cells

**Fig 5:**
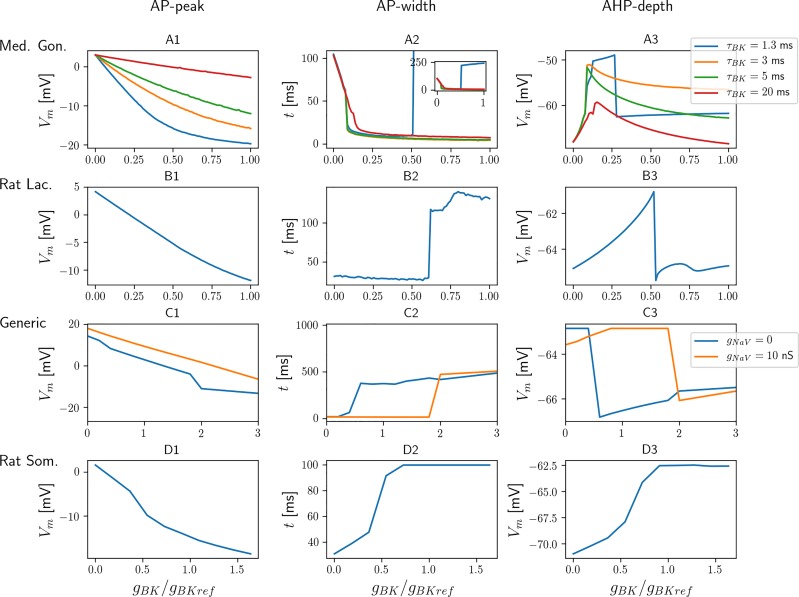
Effects of *I*_*BK*_ on AP shape in different pituitary cell models. (A) Four versions of the medaka gonadotroph model were studied, differing in terms of the BK activation time constant (*τ*_*BK*_). The default parameterization had *τ*_*BK*_ = 3 ms (orange curve). The inset in A2 shows the same curves as the main panel, but with a wider range on the *y*-axis. (B) The rat lactotroph model was taken from [9]. (C) The generic murine pituitary cell model was taken from [32]. Two versions were considered, one with (orange curve) and one without (blue curve) sodium conductances added in the model. (D) The rat somatotroph model was taken from [27]. (A1-E1) The AP-peak voltage decayed monotonically with *g*_*BK*_ in all models. (A2-D2) An increase in *g*_*BK*_ could lead to both a broadening and narrowing of APs, depending on conditions. AP width was defined as the time between the upstroke and downstroke crossings of the voltage midways between −50 mV and the peak potential. (A3-D3) An increase in *g*_*BK*_ could cause both stronger or weaker afterhyperpolarization (AHP), depending on conditions. AHP was defined as the minimum voltage reached between two spikes. (A-D) In all panels, the x-axis showed *g*_*BK*_ relative to a reference value *g*_*BKref*_, which was taken to be the default BK-conductance in the respective models.

As we have seen, *I*_*BK*_ consistently had a narrowing effect on APs in the medaka gonadotroph model, while it has previously been reported to broaden APs in several models based on data from murine pituitary cells [9, 27, 32]. To gain insight into this dual role, we here explore the relationship between *g*_*BK*_ expression and AP shape in four different models, including (i) the medaka gonadotroph model (Fig 5A), (ii) a previously published models of a rat lactotroph (Fig 5B), (iii) a generic pituitary cell model based on data from rats and mice (Fig 5C), and (iv) a model of a rat somatotroph (Fig 5D). In the medaka gonadotroph model, APs were predominantly mediated by *I*_*Na*_, while in the murine models, APs were predominantly mediated by *I*_*Ca*_. Despite several differences, all models contained *I*_*BK*_ and *I*_*K*_, which were the most important ion channels for mediating the AP downstroke.

In all models, an increase in *g*_*BK*_ consistently lead to a reduction of the AP-peak voltage (Fig 5A1–5D1), as one might expect from a hyperpolarizing current. Additional simulations on the medaka gonadotroph model revealed that the magnitude of the reduction depended strongly on the *I*_*BK*_-activation time constant (*τ*_*BK*_). In the default configuration, *τ*_*BK*_ was set to 3 ms (orange curve in Fig 5A1). With a slower *τ*_*BK*_, *I*_*BK*_ activation remained low during the AP upstroke, and its effect on the AP-peak voltage was low (red curve in Fig 5A1). Contrarily, when *τ*_*BK*_ was faster, *I*_*BK*_ activation largely occurred during the AP upstroke, and *I*_*BK*_ then had a larger effect on the AP-peak value (blue curve in Fig 5A1). In general, *I*_*BK*_ could affect both the upstroke (reducing the peak voltage) and downstroke (repolarizing the membrane) of the AP, and the relative contribution to the two phases would depend on the relative timing of *I*_*BK*_ activation and the AP peak.

The effect on *g*_*BK*_ on AP width (Fig 5A2–5D2) and afterhyperpolarization (AHP) depth, defined simply as the minimum voltage reached between two APs (Fig 5A3–5D3), was more complex and less intuitive. To gain an insight into the mechanisms at play, we start by exploring how *g*_*BK*_ affected the AHP depth (Fig 5A3–5D3). The AHP was predominantly due to the joint effect of the two hyperpolarizing currents, *g*_*BK*_ and *g*_*K*_. It may therefore seem counterintuitive that, for low *g*_*BK*_, an increase in *g*_*BK*_ actually decreased the AHP (less hyperpolarization means higher AHP voltages). The explanation lies in the simultaneous effect that *g*_*BK*_ had on reducing the AP-peak voltage (Fig 5A1–5D1). Lower AP-peak values generally meant less *I*_*K*_ activation [9, 25], as this current activates at high voltage levels. Hence, *g*_*BK*_ had a dual affect on the AHP. It could facilitate AHP through its direct, hyperpolarizing effect, but at the same time counteract AHP indirectly, by limiting *g*_*K*_ activation. When *g*_*BK*_ was small, the AHP was predominantly mediated by *g*_*K*_, and the indirect effect dominated, so that an increase in *g*_*BK*_ reduced the AHP up to a certain point, where the direct effect to took over, so that a further increase in *g*_*BK*_ enhanced the AHP.

The dual role of *g*_*BK*_ is also reflected in the effect that *g*_*BK*_ had on the AP width (Fig 5A2–5D2). An increase in *g*_*BK*_ could either lead to briefer APs, through the direct hyperpolarizing effect of *I*_*BK*_, or broader APs, through the indirect effect of *g*_*BK*_ reducing *I*_*K*_ activation. This dual role of *g*_*BK*_ is seen most clearly in the rat lactotroph model (Fig 5B2). For low values of *g*_*BK*_, the direct effect dominated, and the AP width decayed monotonically with increasing *g*_*BK*_ up to a certain threshold value, where a further increase in *g*_*BK*_ gave a sharp transition to very broad APs (pseudo-plateau bursts). The paradoxical role of *I*_*BK*_ as a burst-promoter in the lactotroph model was explored in detail in the original study [9], and in a later re-implementation of the model [37]. The same dual role of *g*_*BK*_ on the AP shape was seen in the generic pituitary model, although the effect of *g*_*BK*_ on narrowing APs for low *g*_*BK*_ was there very small (Fig 5C2). In the rat somatotroph model, the indirect effect dominated for all values of *g*_*BK*_, and the AP width increased monotonically with increasing *g*_*BK*_ (Fig 5D2). Oppositely, in the default parameterization of the medaka gonadotroph model, the direct effect dominated for all values of *g*_*BK*_, and the AP width decreased monotonically with increasing *g*_*BK*_ (orange curve, Fig 5A2). Only by decreasing *τ*_*BK*_ to unrealistically low values, *g*_*BK*_ could have a broadening effect on APs in the medaka gonadotroph model (blue curve, inset in Fig 5A2).

We note that the complex interplay of mechanisms is only partly captured by the simplified, heuristic explanations presented above. In particular, for high *g*_*BK*_ values, neither the AP width or AHP increased monotonically with *g*_*BK*_ in all models (Fig 5B2 and 5C3). This non-monotonic behavior putatively reflects a complex and highly sensitive interplay between several mechanisms active in the aftermath of the AP peak, and we did not attempt to explore it in further detail.

**Fig 6:**
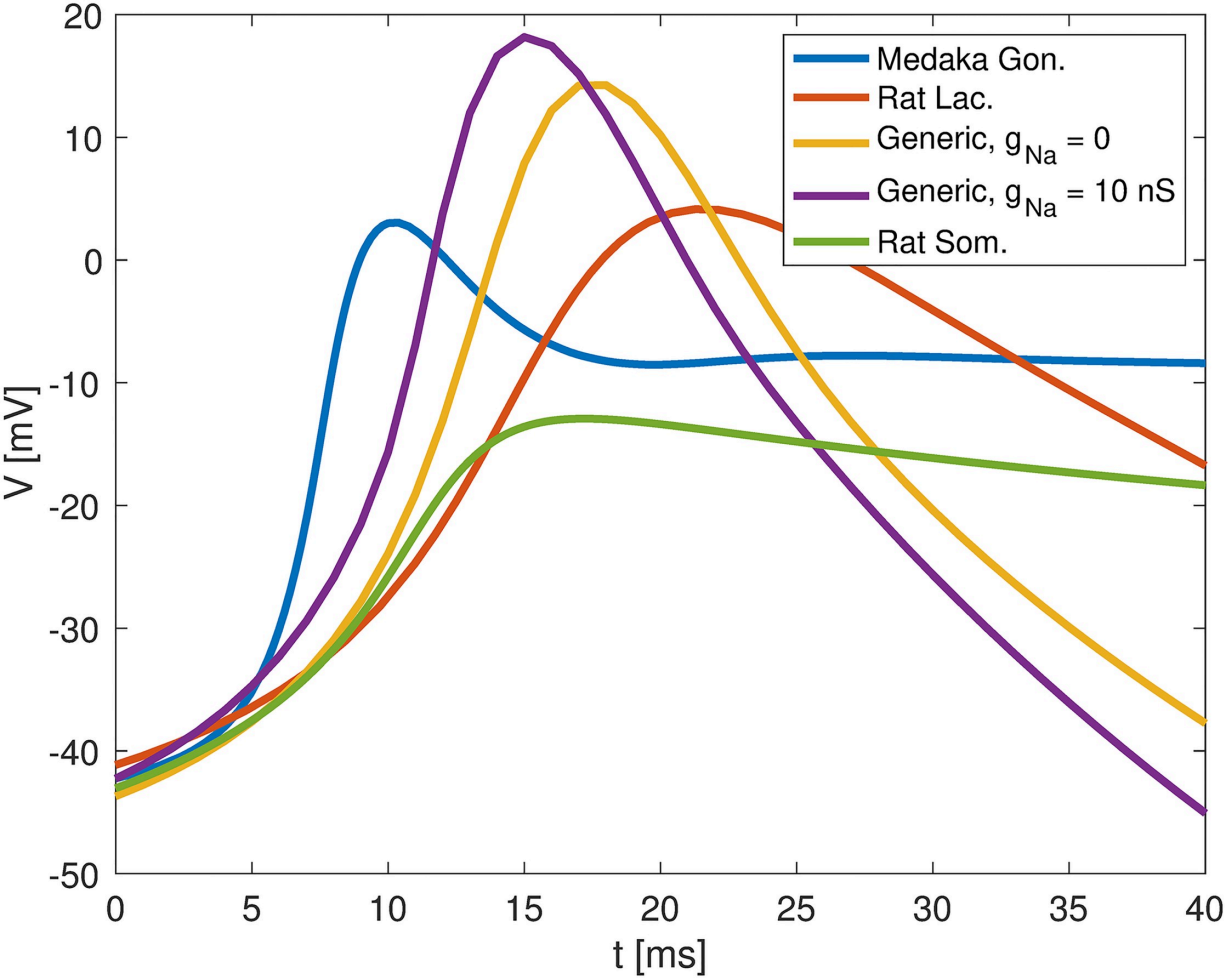
Action potential upstrokes in different pituitary cell models. The AP upstroke was steepest in the medaka gonadotroph model (where it was mediated by *I*_*Na*_), and slower in the murine pituitary models where they were predominantly mediated by *I*_*Ca*_. The models considered were (blue) the default parameterization of the medaka gonadotroph model, (red) the rat lactotroph model, a generic murine pituitary cell model with (purple) and without (orange) sodium conductances, and (green) the rat somatotroph model. In all models, the BK conductance was set to zero.

As we have seen, *I*_*BK*_ facilitated bursting in all the considered models based on murine data, but not in the default parameterization of the medaka gonadotroph model. As the *I*_*BK*_ kinetics in the medaka gonadotroph model was essentially the same as in the generic murine pituitary cell model [32], we hypothesized that the different role played by *I*_*BK*_ in the medaka gonadotroph model versus the murine pituitary cell models was due to differences in AP shape, rather than *I*_*BK*_ kinetics. By comparing the AP upstrokes of the different models, we see that the fastest upstroke was found in the medaka gonadotroph model where APs were predominantly *I*_*Na*_ mediated (blue curve in Fig 6). The second fastest upstroke was seen in the generic murine model in the case where its APs were mediated by a combination of *I*_*Ca*_ and *I*_*Na*_ (purple curve in Fig 6). Hence, the addition of *I*_*Na*_ to this model made the AP upstroke steeper, and, as we saw in Fig 5C2, this made the model less susceptible to bursting, i.e., the transition to bursting occurred for a much higher value of *g*_*BK*_.

In the remaining models, APs were mediated solely by *I*_*Ca*_, and had slower upstroke. Hence, in the murine models, *I*_*BK*_ had more time to activate during the AP upstrokes, which explains why *I*_*BK*_ indirectly could promote bursting in these models, by reducing AP-peak value and thereby *I*_*K*_ activation. In order to have the same effect in the medaka gonadotroph model, *τ*_*BK*_ needed to be speeded up dramatically, as illustrated in the blue curve in Fig 5A2. This scenario is hypothetical, as no experiments suggest that *I*_*BK*_ does promote bursting in medaka gonadotrophs. However, it is interesting to note that this parameterization of the model fired bursts (i.e., APs with width > 60 ms) both for very low and very high values for *g*_*BK*_, while it fired regular AP for intermediate *g*_*BK*_ value. Also a previous model of a rat corticotroph showed such bursting behaviour in two disjoint regions in *g*_*BK*_-space (see Fig 3 in [31]).

In summary, *I*_*BK*_ had an inhibitory effect on *I*_*K*_ by reducing the AP amplitude, and a collaborative effect with *I*_*K*_ in mediating the AP downstroke. With slow AP upstrokes, as in the murine pituitary cell models, the inhibitory effect of *I*_*BK*_ on *I*_*K*_ could dominate, and *I*_*BK*_ could result in a net reduction of hyperpolarization and as such promote broader APs and sometimes bursts. With faster AP upstrokes, such as in the medaka gonadotroph models, the collaborative effect of *I*_*BK*_ always dominated, and *I*_*BK*_ consistently facilitated narrower APs. In a broader scope, this suggests that *I*_*BK*_ can act as a mechanism that reduces the duration of already fast APs, and prolongs the duration of already slow APs.

## Discussion

TTX-sensitive Na^+^ currents (*I*_*Na*_) are present in all pituitary cells, but are in many cases inactive during spontaneous activity [8]. Previous models of the electrical activity of pituitary cells have focused on conditions where *I*_*Na*_ is of lesser importance, and where AP generation is predominantly mediated by high-voltage activated Ca^2+^ currents [3, 9, 23, 25–28]. To our knowledge, we have in the current work presented the first models that describe pituitary cells under conditions where AP generation is *I*_*Na*_-mediated. The model was adapted to experimental data from LH-producing gonadotrophs in medaka, whose spontaneous activity is highly *I*_*Na*_-dependent. Voltage-clamp data was used to develop models for the activation kinetics for *I*_*Na*_ and *I*_*Ca*_ currents, and the firing properties of the model were further adapted to current-clamp data from spontaneously active cells (under control conditions, and after application of TTX and paxilline).

To examine the consequences of having different AP generation mechanisms, we performed a comparison between the the medaka gonadotroph model, which fired *I*_*Na*_-mediated APs, and three models of murine pituitary cells which fired APs that were exclusively mediated by *I*_*Ca*_ [9, 27], or by a combination of *I*_*Ca*_ and *I*_*Na*_ [32]. The most interesting result that came out of this comparison was that *I*_*BK*_ had a dual role on AP shape, and could under some conditions broaden APs and promote bursting, and under other conditions make them narrower. We suggested that the broadening effect could only occur in scenarios where *I*_*BK*_ had sufficient time to activate during the AP upstroke, and thus required either a very fast *I*_*BK*_ activation time constant, or a relatively slow AP upstroke. In the murine models, the AP upstrokes were slower than in the medaka gonadotroph model, and we suggest that this explains why increased *I*_*BK*_ can promote bursting in many murine pituitary cells [8, 9, 25, 32], but not in medaka gonadotrophs [12]. Also other K^+^ channels have been shown to have such a burst-promoting role in murine pituitary cells [38, 39]. It should be noted that the effect on reducing AP width is a commonly reported role for *I*_*BK*_ in many excitable cells [40–44], while the AP-broadening and burst promoting effect that *I*_*BK*_ is less conventional.

The role of *I*_*BK*_ as a burst promoter has not been found consistently in rat lactotrophes. In the study by Miranda et al. 2003, AP width in rat GH_3_, a widely used model for pituitary lactotrophs, was instead found to increase when *I*_*BK*_ was blocked with paxilline [43], i.e., similar to what we found for medaka gonadotrophs (Fig 1D). The different effects of *I*_*BK*_ on AP width observed in different laboratories [25, 40, 43] was addressed by Tabak et al. 2011 [9], who proposed possible explanations that could reconcile the conflicting results. One possible explanation could be there is a variability in terms of how BK channels are localized in various cells, and that BK channels that are co-localized with Ca^2+^ channels will respond rapidly to voltage fluctuations and promote bursting, while BK channels that are not co-localized with Ca^2+^ channels will react more slowly to voltage fluctuations and have the opposite effect [9]. A second possible explanation, also suggested by Tabak et al. 2011, was that *I*_*BK*_ might have different kinetic properties in different cells due to variations in their phosphorylation state [9]. A third explanation could be that different cells have different BK splice variants [45], or different regulatory sub-units.

The model comparison in Fig 5 provides additional possible explanations to the conflicting conclusions regarding the role of *I*_*BK*_ in lactotrophs. Firstly, the fact that *I*_*BK*_ has affects the AP shape differently in different cells does not by necessity reflect differences in *I*_*BK*_ kinetics or localization. Simulations on the rat lactotroph model showed that the same *I*_*BK*_ could both have a broadening and narrowing effect on the APs within one and the same model (Fig 5B). That is, APs could be made broader either by reducing *g*_*BK*_ to very low values, or by increasing it to very high values. This dual effect of *I*_*BK*_ was even more pronounced in a version of the medaka gonadotroph model (blue curve in Fig 5B), and a previous model of rat corticotrophs [31], which elicited bursts both under full *g*_*BK*_ blockage and for large *g*_*BK*_ expression, while they fired regular APs for intermediate *g*_*BK*_ expression. Hence, in general, *g*_*BK*_ blockage could affect the AP width in either way, depending on the initial level of *g*_*BK*_ expression, and conflicting conclusions regarding the role of *I*_*BK*_ could reflect that variations in *g*_*BK*_ expression under control conditions. Secondly, the way on which *I*_*BK*_ will affect the APs in a given cell can not be predicted from *g*_*BK*_ kinetics/expression alone, but also depends on the AP generating mechanisms in the cell. Our simulations suggested that APs with a steep upstroke were prone to be made briefer by *I*_*BK*_, while APs with a slower upstroke were prone to be prolonged by *I*_*BK*_, as also suggested in the dynamic clamp experiments by Tabak et al. [9]. Putatively, *I*_*Na*_ mediated APs will generally have a steeper upstroke than *I*_*Ca*_ mediated APs, as was the case in the models studied here. If this is the case, the role of *I*_*BK*_ in a given cell may thus largely be determined by which membrane mechanisms that mediate its AP upstroke, and especially the degree to which *I*_*Na*_ is involved, which is highly resting-potential dependent, and likely to depend strongly on experimental conditions. Differences in *I*_*Na*_ involvement could in principle explain the conflicting experiments on rat lactotrophs [25, 43]. In the experiment by Van Goor et al. 2001, where *I*_*BK*_ was found to broaden APs, APs were predominantly mediated by *I*_*Ca*_ [9, 25]. In the experiment by Miranda et al. 2003, where *I*_*BK*_ was found to narrow APs (i.e., blocking *I*_*BK*_ lead to broader APs), it was reported that this *only* occurred under conditions in which short APs were present. It is likely that the events described in that work as *short APs* were largely *I*_*Na*_-mediated, so that the differences between the studies by Van Goor et al. 2011 and Miranda et al. 2003 suggest that different AP generation mechanisms dominated in the two experiments.

Although the medaka gonadotroph model captured the essential firing properties of medaka gonadotrophs, the agreement between model and data was not perfect. For example, the AP width during control conditions (Fig 3A2) was in the upper range of that seen in the experiments, while AP peak voltage was in the lower range of what was seen in the experiments (Fig 1A and 1B). We were not able to obtain briefer APs with larger peak values without compromising the agreement between the experimental data and other model features, such as afterhyperpolarization, firing rate and response to *I*_*BK*_-blockage. The conductances selected for the default model (Table 1) were thus a compromise made to obtain an acceptable match to several features simultaneously. The fact that we were not able to obtain a more accurate match between model and data likely reflect that some of the ion channels present in the model are imperfect representations of the ion channels present in the real cell. For example, the simplified kinetics schemes used for *I*_*K*_, *I*_*BK*_ and *I*_*SK*_ were adopted from models of rat pituitary cells [9, 32], and were not constrained to data from medaka gonadotrophs. In addition, the biological cell is likely to contain a variety of additional ion channels that were not included in the model.

To our knowledge, the medaka gonadotroph model is the first computational model of an endocrine cell that fires APs that are predominantly *I*_*Na*_-based. Although it was adapted to experimental recordings from LH-producing gonadotrophs in medaka, we believe that the model has a more general value. Different types of pituitary cells in several different species share many of the same membrane mechanisms [8]. In particular, *I*_*Na*_-based APs are elicited by several pituitary endocrine cell types and in several animal species, depending on biological conditions [4, 7, 17, 18, 33–35]. It is thus likely that the response patterns of related cell types may be captured by up- or down-regulation of selected mechanisms included in medaka gonadotroph model. Insight in which parameters that should be adjusted in order to obtain desired changes in the model’s firing properties could then be obtained through a sensitivity analysis, such as that presented in Fig 4, or in previous, more comprehensive studies that consider a larger number of model features [37, 39].

## Methods

### Experimental procedures

The electrophysiological experiments were conducted using the patch-clamp technique on brain-pituitary slices from adult female medaka (as described in [46]). To record spontaneous action potentials and Ca^2+^ currents we used amphotericin B perforated patch configuration, while for Na^+^ currents we used whole cell configuration. Extracellular (EC) solution used for recording spontaneous action potentials (current clamp) contained 134 mM NaCl, 2.9 mM KCl, 2 mM CaCl_2_, 1.2 mM MgCl_2_ 1.2, 10 mM HEPES, 4.5 mM glucose. The solution was adjusted to a pH of 7.75 with NaOH and osmolarity adjusted to 290 mOsm with mannitol before sterile filtration. Before use, the EC solution was added 0.1% bovine serum albumin (BSA). For Na^+^ current recordings (voltage clamp) we used a Ca^2+^ free and Na^+^ fixed (140 mM) EC solution, pH adjusted with trizma base. In addition, 10 *μ*M nifedipine, 2 mM 4-Aminopyridine (4-AP) and 4 mM Tetraethylammonium (TEA) was added to the EC solution just before the experiments. To record Ca^2+^ currents, we substituted NaCl with 120 mM choline-Cl and added 20 mM Ca^2+^, 2 mM 4-AP and 4 mM TEA. The patch pipettes were made from thick-walled borosilicate glass using a horizontal puller (P 100 from Sutter Instruments). The resistance of the patch pipettes was 4-5 MΩ for perforated patch recordings and 6-7 MΩ for whole-cell recordings. For recordings of spontaneous action potentials, the following intracellular (IC) electrode solution was added to the patch pipette: 120 mM KOH, 20 mM KCl, 10 mM HEPES, 20 mM Sucrose, and 0.2 mM EGTA. The pH was adjusted to 7.2 using C^6^H^13^NO^4^S (mes) acid, and the osmolality to 280 mOsm using sucrose. The solution was added 0.24 mg/ml amphotericin B to perforate the cell membrane (see [46] for details). In voltage clamp experiments the K^+^ was removed from the intracellular solution to isolate Na^+^ and Ca^2+^ currents. This was achieved by substituting KOH and KCl with 130 mM Cs-mes titrated to pH 7.2 with CsOH. The electrode was coupled to a Multiclamp 700B amplifier (Molecular Devices) and recorded signal was digitized (Digidata 1550 with humsilencer, Molecular Devices) at 10 KHz and filtered at one-third of the sampling rate. In selected experiments, voltage-gated Na^+^ channels were blocked using 5 *μ*M TTX, and BK channels were blocked using 5 *μ*M paxilline. Both drugs were dissolved in EC solution and applied using 20 kPa puff ejection through a 2 MΩ pipette, 30-40 *μ*m from the target cell.

Under the experimental (voltage-clamp) conditions used for recording Na^+^ currents, and under the experimental current-clamp conditions, a liquid junction potential of about −9 mV was calculated and corrected for in the data shown in Fig 1, and in the kinetics model for *I*_*Na*_ (Fig 2A). A liquid junction potential of about −15 mV was calculated for the experimental (voltage-clamp) conditions used for recording Ca^2+^ currents, and was corrected for in the kinetics model for $I_{Ca}^{m}$ (Fig 2B).

### Ethics statement

Handling, husbandry and use of fish were in accordance with the guidelines and requirements for the care and welfare of research animals of the Norwegian Animal Health Authority and of the Norwegian University of Life Sciences.

### Model of medaka gonadotroph

As stated in the Results-section, the medaka gonadotroph model was described by the equation:

$$\begin{array}{c} C_{m}\frac{dV}{dt}=-\left(I_{Na}+I_{Ca}+I_{K}+I_{BK}+I_{SK}+I_{leak}\right). \end{array}$$ (2)

The membrane capacitance was set to the standard value *C*_*m*_ = 1*μ*F/cm^2^, and the leak conductance was described by

$$\begin{array}{c} I_{leak}=g_{leak}\left(V-E_{leak}\right). \end{array}$$ (3)

with a reversal potential *E*_*leak*_ = −45 mV. Due to a nonzero-activation of *I*_*K*_ around the resting level, this gave an effective resting potential around −50 mV, similar to that in the experimental data in Fig 1.

The kinetics of all ion channels were summarized in Fig 2, but are described in further detail here. *I*_*Na*_ was modeled using the standard Hodgkin and Huxley-form [47]:

$$\begin{array}{c} I_{Na}=g_{Na}q^{3}h\left(V-E_{Na}\right), \end{array}$$ (4)

with a reversal potential *E*_*Na*_ = 50 mV, and gating kinetics defined by:

$$\begin{array}{c} \frac{dq}{dt}=\frac{q_{\infty }-q}{\tau_{q}},\frac{dh}{dt}=\frac{h_{\infty }-h}{\tau_{h}}. \end{array}$$ (5)

The steady-state activation and time constants (*q*_∞_, *h*_∞_, *τ*_*q*_ and *τ*_*h*_) were fitted to voltage-clamp data from medaka gonadotrophs, as described below, in the subsection titled “Model for the voltage-gated Na^+^ channels”.

*I*_*Ca*_ was modelled using the Goldman-Hodgkin-Katz formalism, which accounts for dynamics effect on Ca^2+^ reversal potentials [48]:

$$\begin{array}{c} I_{Ca}^{m}=g_{Ca}m^{2}\frac{F^{2}}{RT}V\frac{\left[Ca\right]-{\left[Ca\right]}_{e}\text{exp}\left(\frac{-VF}{RT}\right)}{1-\text{exp}(\frac{-VF}{RT})}, \end{array}$$ (6)

with

$$\begin{array}{c} \frac{dm}{dt}=\frac{m_{\infty }-m}{\tau_{m}} \end{array}$$ (7)

Here, *R* = 8.314J/(mol ⋅ K is the gas constant, *F* = 96485.3C/mol is the Faraday constant, *T* is the temperature, which was set to 293.15 K in all simulations. [*Ca*] and [*Ca*]_*e*_ were the cytosolic and extracellular Ca^2+^ concentrations, respectively. The former was explicitly modelled (see below), while the latter was assumed to be constant at 2 mM. As Eq 6 shows, we used two activation gates *m*. This is typical for models of L-type Ca^2+^ channels (see e.g. [49–52]), which are the most abundantly expressed HVA channels in the cells studied here [11]. The steady-state activation and time constant (*m*_∞_ and *τ*_*h*_) were fitted to voltage-clamp data from medaka gonadotrophs, as described below, in the subsection titled “Model for high-voltage activated Ca^2+^ channels”. We note that *g*_*Ca*_ in the Goldman-Hodgkin-Katz formalism (Eq 6) is not a conductance, but a permeability with units cm/s. It is proportional to the conductance, and for simplicity, we have referred to it as a conductance in the text.

The delayed rectifyer K^+^ channel was modelled as

$$\begin{array}{c} I_{K}=g_{K}n\left(V-E_{K}\right), \end{array}$$ (8)

with reversal potential *E*_*K*_ = −75 mV, and a time dependent activation gate described by

$$\begin{array}{c} \frac{dn}{dt}=\frac{n_{\infty }-n}{\tau_{K}}. \end{array}$$ (9)

The steady-state activation was described by:

$$\begin{array}{c} n_{\infty }={\left[1+exp\left(\left(v_{n}-V\right)/s_{n}\right)\right]}^{-1}. \end{array}$$ (10)

with a slope parameter *s*_*n*_ = 10 mV, and half-activation *v*_*n*_ = −5 mV. The model for *I*_*K*_ was identical to that in a previous rat lactotroph model [9], with the exception that the constant (voltage-independent) time constant *τ*_*K*_ was made faster (5 ms) in the medaka gonadotroph model to account for the more rapid APs elicited by these cells.

The model for the BK-channel kinetics was a was taken from a previous model (where it was called BK-near) of murine corticotrophs [29, 30], which has also been used in a generic rat pituitary cell model [32]:

$$\begin{array}{c} I_{BK}=g_{BK}f\left(V-E_{K}\right). \end{array}$$ (11)

The activation kinetics was given by:

$$\begin{array}{c} \frac{df}{dt}=\frac{f_{\infty }-f}{\tau_{BK}}, \end{array}$$ (12)

The constant (voltage-independent) activation time constant *τ*_*BK*_ was set to 3 ms. The steady-state activation was given by [29]:

$$\begin{array}{c} f_{\infty }={\left[1+exp\left(\left(v_{f}-V\right)/3\right)\right]}^{-1}, \end{array}$$ (13)

with

$$\begin{array}{c} v_{f}=0.1-18\cdot \log\left(c_{dom}/c_{ref}\right) \end{array}$$ (14)

As BK channels are often co-localized with high-voltage activated Ca^2+^ channels, BK activation was assumed to depend on a domain concentration *c*_*dom*_ in Ca^2+^ nanodomains, which in turn was assumed to be proportional to the instantaneous Ca^2+^ influx through *I*_*Ca*_. We therefore set:

$$\begin{array}{c} c_{dom}=-AI_{Ca}, \end{array}$$ (15)

where *A* = 1.21 mmol⋅cm^−1^⋅C^−1^ is a parameter that converts a current density into a concentration, and *c*_*ref*_ = 2*μM* is a reference concentration. The parameter *A* was not taken from previous studies, but tuned so that the model obtained suitable BK activation in simulations on the medaka gonadotroph model.

Finally, the SK channel was the same as in the previous model of a rat lactotroph [9], and was modelled as:

$$\begin{array}{c} I_{SK}=g_{SK}s_{\infty }\left(\left[Ca\right]\right)\left(V-E_{K}\right), \end{array}$$ (16)

with an instantaneous, Ca^2+^ dependent, steady-state activation:

$$\begin{array}{c} s_{\infty }\left(\left[Ca\right]\right)=\frac{{\left[Ca\right]}^{2}}{{\left[Ca\right]}^{2}+k_{s}^{2}} \end{array}$$ (17)

where [*Ca*] denotes the cytosolic Ca^2+^ concentration, and *k*_*s*_ is a half-activation concentration of 0.4 *μ*M.

*I*_*Ca*_ and *I*_*SK*_ were dependent on the global cytosolic Ca^2+^ concentration. This was modelled as a simple extrusion mechanism, receiving a source through *I*_*Ca*_, and with a concentration dependent decay term assumed to capture the effects of various ion pumps and buffering mechanisms:

$$\begin{array}{c} \frac{d[Ca]}{dt}=-f_{c}\left(\alpha I_{Ca}+k_{c}\left[Ca\right]\right). \end{array}$$ (18)

Here, *f*_*c*_ = 0.01 is the assumed fraction of free Ca^2+^ in the cytoplasm, *α* = 0.015mM ⋅ cm^2^/*μ*C converts an incoming current to a molar concentration, and *k*_*c*_ = 0.12ms^−1^ is the extrusion rate [9].

The conductances used in the default parameterization of the model are given in Table 1.

### Model for the voltage-gated Na^+^ channels

**Fig 7:**
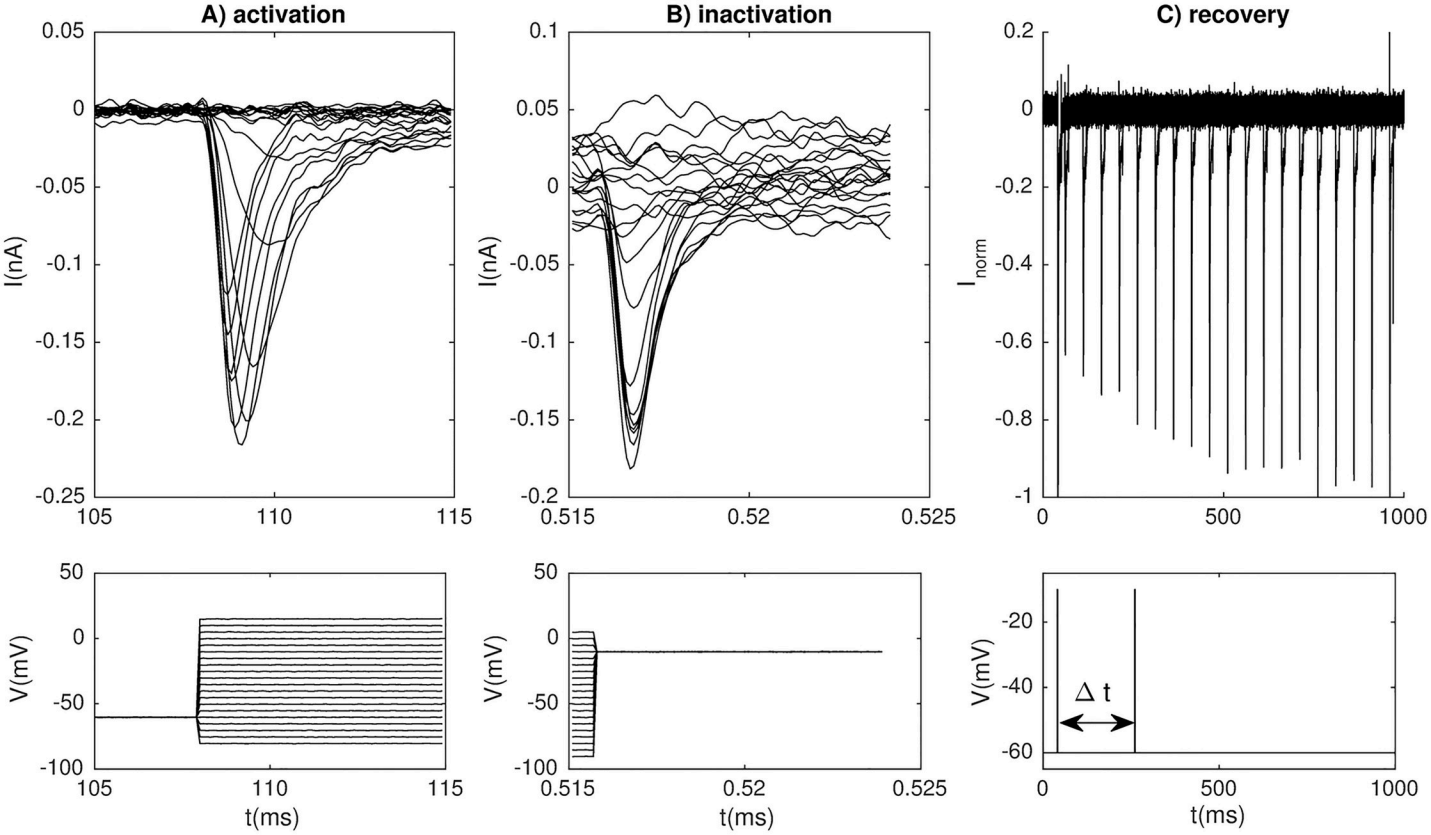
Experimentally recorded Na^+^ currents. (A) Na^+^ currents evoked by the activation protocol. (B) Na^+^ currents evoked by the inactivation protocol. (C) Na^+^ currents used to determine the time constant for recovery from inactivation. (A-C) Voltage protocols are shown below the recorded currents, and all panels show a series of experiments (traces). In (C), the cell was exposed to a pair of square 10 ms pulses arriving with various inter-pulse intervals (Δ*t*). The first pulse always arrived after 40 ms (and coincided in all experiments), while each secondary spike represents a specific experiment (i.e., a specific Δ*t*). The traces were normalized so that the first spike had a peak value of −1 (corresponding to approximately −0.25 pA).

The steady-state values and time courses of the gating kinetics were determined using standard procedures (see e.g. [35, 47, 53, 54]), and was based on the experiments summarized in Fig 7. To determine activation, the cell was held at −60 mV for an endured period, and then stepped to different holding potentials between −80 to 100 mV with 5 mV increments (Fig 7A2), each for which the response current (*I*_*Na*_) was recorded (Fig 7A1). The inactivation properties of Na^+^ were investigated using stepwise pre-pulses (for 500 ms) between −90 and 55 mV with 5 mV increments before recording the current at −10 mV (Fig 7B2). The resulting Na^+^ current then depended on the original holding potential (Fig 7B1). Finally, the recovery time for the Na^+^ current was explored by exposing the cell to a pair of square pulses (stepping from a holding potential of −60 mV to −10 mV for 10 ms) separated by a time interval Δ*t* (Fig 7C2). The smallest Δ*t* was 10 ms, and after this, Δ*t* was increased with 100 ms in each trial. The cell responded to both pulses by eliciting Na^+^-current spikes (Fig 7C2). When Δ*t* was small, the peak voltage of the secondary spike was significantly reduced compared to the first spike, and a full recovery required a Δ*t* in the order of 1/2-1 s.

#### Steady-state activation and inactivation

To determine steady-state activation, the peak current (*I*_*max*_) was determined for each holding potential in Fig 7A, and the maximum peak was observed at about −10 mV. For inactivation, the peak current (*I*_*max*_) was recorded for each holding potential in Fig 7B. In both cases, the maximal conductance (*g*_*max*_) for each holding potential was computed by the equation:

$$\begin{array}{c} g_{max}=I_{max}/\left(V_{hold}-E_{Na}\right). \end{array}$$ (19)

**Fig 8:**
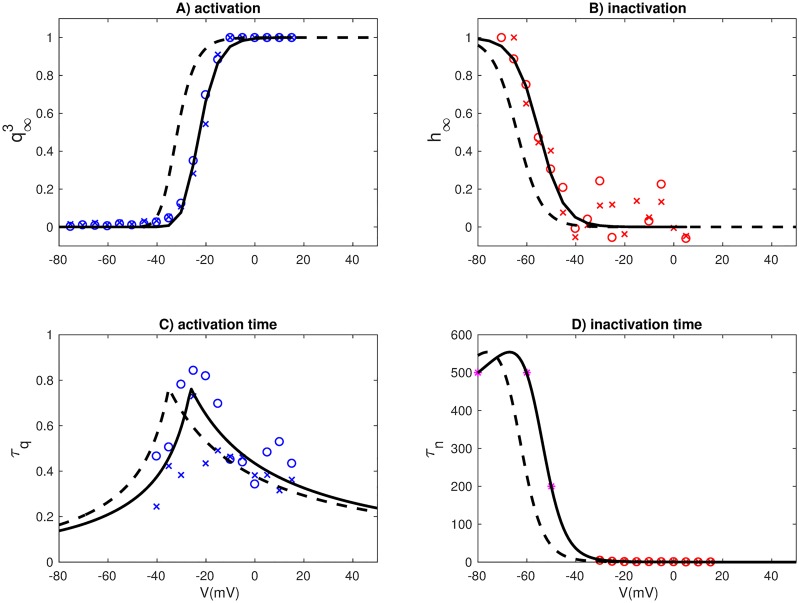
Fitted kinetics for the Na^+^ current. Voltage dependence of steady-state activation (A), steady-state inactivation (B), activation time constant (C) and inactivation time constant (D). The data points and curves in (A-B) were normalized so that activation/inactivation curves had a maximum value of 1 (assuming fully open channels). Dashed lines represent the same model when corrected for a liquid junction potential of 9 mV.

Under the experimental conditions, the intra- and extracellular Na^+^ concentrations were 4 mM and 140 mM, respectively, and the temperature was 26 degrees Celsius, which gives a reversal potential (*E*_*Na*_ = *RT*/*F* ⋅ ln([Na]_ex_)/ln([Na]_in_) of 92 mV. The estimates of *g*_*max*_ for activation and inactivation are indicated by the markers ‘x’ in Fig 8A and 8B, respectively, and markers ‘o’ indicate a second experiment. The dependency of *g*_*max*_ on *V*_*hold*_ was fitted by a Boltzmann curve:

$$\begin{array}{c} f_{bz}=\frac{{\bar{g}}_{max}}{1-\text{exp}{\left(\left[V_{*}-V_{hold}\right)/k\right]}^{a}}, \end{array}$$ (20)

where ${\bar{g}}_{max}$ corresponds to *g*_*max*_ estimated for the largest peak in the entire data set (i.e. at about −0.22 nA in Fig 7A and −0.18 nA in Fig 7B). The factor *k* determines the slope of the Boltzman curve, the exponent *a* corresponds to the number of activation or inactivation gates, and V_*_ determines the voltage range where the curve rises. When *a* = 1 (as for inactivation), V_*_ equals *V*_1/2_, i.e. the voltage where *f*_*bz*_ has reached its half-maximum value. With a higher number of gates, V_*_ = *V*_1/2_ + *k* ⋅ ln(2^1/*a*^ − 1). Eq 20 gave a good fit for the steady state activation $m_{\inf}^{3}$ (Fig 8A) and the steady state inactivation *h*_inf_ (Fig 8B) with the parameter values for *a*, *k* and V_*_ listed in Table 2.

**Table 2:** Parameters for Na^+^ activation. The parameters *p*1-*p*6 are used together with Eq 22 to yield the time constants for steady state activation and inactivation (in units of ms). The remaining parameters are used together with Eq 20 to obtain the steady-state activation and inactivation functions. The curves obtained in this way describe the voltage dependence under experimental conditions, and was afterwards corrected by subtracting the liquid junction potential of −9 mV (see Fig 2A).

parameter	activation value	inactivation value	unit
*p*_1_	0.038	0.040	(ms⋅mV)^−1^
*p*_2_	-60.3	-32.4	mV
*p*_3_	5.77	3.29	mV
*p*_4_	0.135	2.65	(ms⋅mV)^−1^
*p*_5_	-26.17	-2145	mV
*p*_6_	3e-5	139.3	mV
V_*_	28.84	55.0	mV
*k*	4.55	5.07	mV
*a*	3	1	
*V*_*lj*_	-9	-9	mV

#### Time constants for activation and inactivation

With three opening gates (*q*) and one closing gate (*h*), the time constants for activation and inactivation were derived by fitting the function [47]

$$\begin{array}{c} I_{max}=g_{Na}\left(V_{hold}-E_{Na}\right)[1-\text{exp}{\left(1-t/\tau_{q}\right)}^{3}\left(1-\text{exp}\left(1-t/\tau_{h}\right)\right], \end{array}$$ (21)

to the response curves in Fig 7A1. For low step potentials (< −40 mV), the response was too small and noisy to reveal any clear trend, and we were unable to obtain meaningful fits using the functional form of Eq 19. For this reason, only the experiments with a step potential of −40 mV and higher were used when fitting the time constants. The fitting procedure resulted in a pair of time constants (*τ*_*q*_ and *τ*_*h*_) for each step potential in the protocol, as indicated by the data points (‘x’ and ‘o’) in Fig 8C and 8D. The data points obtained by fitting Eq 21 to the traces in Fig 7A1 were sufficient to obtain a clear picture of the voltage dependence of the activation time constant (*τ*_*q*_), which had a peak value at −24 mV, i.e. within the voltage-range for which there was suitable data (Fig 8C). The inactivation time constant (*τ*_*h*_) was, however, monotonously decreasing over the voltage range for which there was good data. We therefore needed additional data points for the voltage dependence of *τ*_*h*_ in the range *V* < −40 mV. Based on the insight from the recovery-experiments (Fig 7C), we expected inactivation to be very slow at the resting potential and below. To account for this, we introduced the three additional data points marked by ‘*’ in Fig 8D, which assure a recovery time in the correct order of magnitude.

The data points for the time constants were fitted with curves on the functional form proposed by Traub et al. [55]:

$$\begin{array}{c} \tau ={\left(\frac{p_{1}\left(p_{2}-V\right)}{\text{exp}\left[\left(p_{2}-V\right)/p_{3}\right]-1}+\frac{p_{4}\left(V-p_{5}\right)}{\text{exp}\left[\left(V-p_{5}\right)/p_{6}\right]-1}\right)}^{-1}. \end{array}$$ (22)

Good fits to the data points were obtained with the parameter values in Table 2.

### Model for high-voltage activated Ca^2+^ channels

When estimating the steady-state values and time constant we followed procedures inspired from previous studies of L-type Ca^2+^ channel activation, we did not use Eq 6, but used the simpler kinetics scheme *I*_*Ca*_ = *g*_*HV A*_*m*^2^(*V* − *E*_*Ca*_) (see e.g. [50])) assuming a constant reversal potential.

#### Steady-state activation

**Fig 9:**
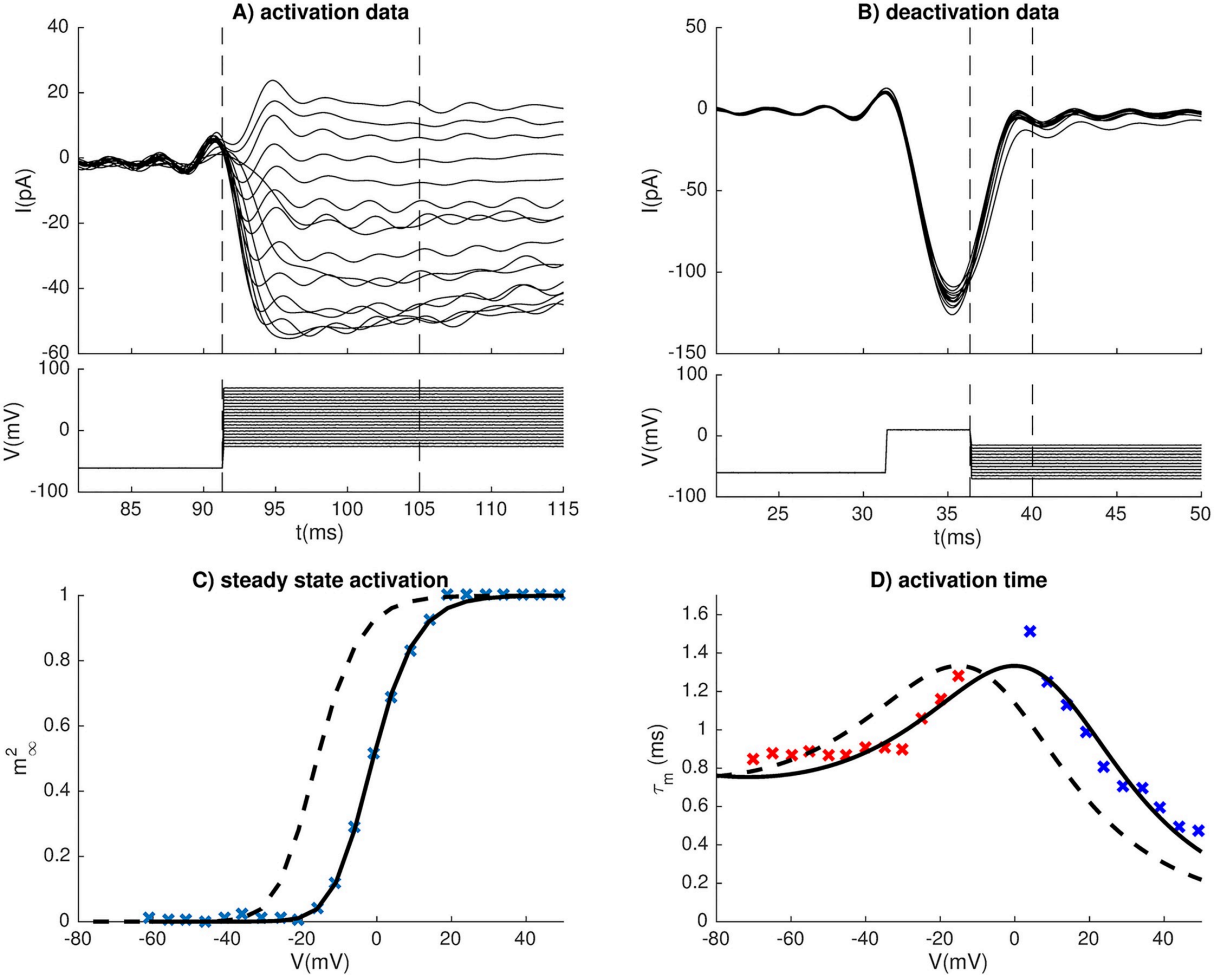
Fitted kinetics for the Ca^2+^ current. (A) *I*_*Ca*_ evoked by the activation protocol. (B) *I*_*Ca*_ evoked by the deactivation protocol. (A-B) Voltage protocols are shown below the recorded currents, and all panels show a series of experiments (traces). The current-traces were low-pass filtered with a cutoff-frequency of 300 Hz. (C) Voltage dependence of steady-state activation, normalized so that the activation curve had a maximum value of 1 (assuming fully open channels). (D) Activation time constant. Red data points were estimated from the deactivation protocol (B), while blue data points were estimated using the activation protocol (A). Dashed lines in (C-D) denote the kinetics scheme when corrected for a liquid junction potential of −15 mV for the experimental conditions used for recording Ca^2+^ currents.

The steady-state value and time constant for *m* were determined from the experiments summarized in Fig 9A and 9B. To study steady-state activation, the cell was held at −60 mV for an endured period, and then stepped to different holding potentials, each for which the response current (*I*_*Ca*_) was recorded (Fig 9A). Due to the small cellular size, perforated patches was used for recording the Ca^2+^ currents, and the recorded currents were small and noisy. As Fig 9A shows, the *I*_*Ca*_ responses did not follow a characteristic exponential curve towards steady state, as seen in many other experiments. Likely, this was due to *I*_*Ca*_ comprising a complex of different HVA channels (e.g., P, Q, R, L-type) which have different activation kinetics [49–51, 56–58]. In addition, some in some of the weaker responses *I*_*Ca*_ even switched from an inward to an outward current, something that could indicate effects of ER release on the calcium reversal potential. Due to these complications, only the early part of the response was used, i.e., from stimulus onset and to the negative peak value in interval indicated by dashed vertical lines). Voltage-dependent deactivation of Ca^2+^ currents (Fig 9B) was examined by measuring the tail current that followed after a 5 ms step to 10 mV when returning to voltages between −10 mV and −60 mV. The deactivation protocol was used to provide additional data points for the activation time constants (see below).

The peak current (*I*_*max*_) was recorded for each holding potential in Fig 9A, and the maximum peak was observed at about 20 mV. By observations, (*I*_*Ca*_) became an outward current when step potentials were increased beyond 70 mV, and based on this we assumed a reversal potential of *E*_*Ca*_ = 70 mV. Similar to what we did for the Na^+^ channel, the maximal conductance (*g*_*max*_) for each holding potential was computed by the equation:

$$\begin{array}{c} g_{max}=I_{max}/\left(V_{hold}-E_{Ca}\right). \end{array}$$ (23)

The estimates of *g*_*max*_ for activation are indicated by the crosses in Fig 9C. The dependency of *g*_*max*_ on *V*_*hold*_ was then fitted by a Boltzmann curve (Eq 20), with *a* = 2 activation gates, and a good fit was obtained with values for *a*, *k* and V_*_ as in Table 3.

**Table 3:** Parameters for Ca^2+^ activation. The parameters *p*1-*p*6 are used together with Eq 22 to yield the time constants for steady state activation (in units of ms). The remaining parameters are used together with Eq 20 to obtain the steady state activation function. The curves obtained in this way describe the voltage dependence under experimental conditions, and was afterwards corrected with a liquid junction potential of −15 mV (see Fig 2B).

parameter	activation value	unit
*p*_1_	-0.128	(ms⋅mV)^−1^
*p*_2_	-46.7	mV
*p*_3_	19.0	mV
*p*_4_	-101.54	(ms⋅mV)^−1^
*p*_5_	535.1	mV
*p*_6_	-60.0	mV
V_*_	-6.79	mV
*k*	6.57	mV
*a*	2	
*V*_*lj*_	-15	mV

#### Time constants for *I*_*Ca*_ activation

Assuming two opening gates (*m*), the time constant for *I*_*Ca*_-activation was derived by fitting the function [47]:

$$\begin{array}{c} I_{max}=g_{Ca}\left(V_{hold}-E_{Ca}\right)\left[1-\text{exp}{\left(1-t/\tau_{m}\right)}^{2}\right] \end{array}$$ (24)

to the response curves in Fig 9A and 9B. The activation protocol was used to determine *τ*_*m*_ at high step potentials (from −5 mV and upwards), where the response was not too small and noisy to reveal any clear trend (blue data points in Fig 9D). The deactivation protocol was used to determine *τ*_*m*_ for lower step potentials (red data points in Fig 9D). Like for the Na^+^ channel, the voltage dependence of the time constants were fitted using the functional form in Eq 22. Good fits to the data points were obtained with the parameter values in Table 3.

### Implementation details

#### Software

The medaka gonadotroph model and the rat lactotroph model [9] were implemented using the Python interface for the NEURON simulator [59]. For the rat lactotroph model, we used a previous implementation [37]. All simulations on these two models were run using adaptive time stepping provided by the NEURON simulator. The generic murine pituitary cell model was taken from the supplementary data in the original pulblication [32], and the rat somatotroph model [27] was downloaded from https://lbm.niddk.nih.gov/sherman/gallery/neural/somatotroph//figure4.ode. These models were coded in XPP [60], and we exported data from XPP simulations and analyzed them in Matlab to obtain the metrics plotted in Fig 5.

Experimental current-clamp data (Fig 1), experimental voltage-clamp data (Figs 1, 7, 8, and 9)) and fitted ion-channel kinetics (Fig 2) were plotted using Matlab (http://se.mathworks.com/). When comparing AP shapes, simulations performed in XPP and NEURON were exported to Matlab arrays, and plotted in Matlab (Fig 6). All other plots were made in Python (http://www.python.org).

The sensitivity analysis (Fig 4) was performed by aid of the Python-based toolbox Uncertainpy [36]. The features considered (*IsBursting*, *IsRegular*, and *IsNotSpiking* were custom made for the analysis in the current work. Uncertainpy was run using polynomial chaos with the point collocation method (the default of Uncertainpy) and a polynomial order of five. The sensitivity analysis was based on calculating Sobol indices. Only the total-order Sobol indices were presented. A total-order Sobol index quantifies the sensitivity of a feature to a given parameter, accounting for all higher order co-interactions between the parameter and all other parameters (see [61] or the brief overview in Appendix B of [62]). For the sensitivity analysis (Fig 4), the model was run for 60,000 ms, and the first 10,000 ms were discarded to eliminate initial transients, while the remaining 50,000 ms were used in the uncertainty analysis.

The medaka gonadotroph model, and the code for generating Figs 3 and 4 are available for download (doi: 10.5281/zenodo.3359635).

#### Construction and tuning of the medaka gonadotroph model

The model construction and tuning of the medaka gonadotroph model were done in several steps. First, we took the membrane capacitance (*C*_*m*_), the models from *I*_*leak*_, *I*_*K*_ and *I*_*SK*_, and the reversal potentials for *E*_*Na*_ and *E*_*K*_ from the previous model of a rat lactotroph [9], and implemented them in the Python interface for the NEURON simulator [59]. As a starting point, we used the same parameterization as the original model [9]. However, in the original model, *C*_*m*_ and the conductances (*g*_*X*_) were given as a total capacitance and total conductances, and the implementation in NEURON required that we converted them from total currents/capacitance to currents/capacitance per membrane area. To do this, we *divided* them by scaling factor

$$\begin{array}{c} F_{scale}=10.05\cdot 10^{-6}\;{\text{cm}}^{2}, \end{array}$$ (25)

which was selected so that the final capacitance per membrane area equal to 1*μ*F/cm^2^. The same scaling was used when adopting *I*_*BK*_ from [30]. Furthermore, the internal calcium handling (Eq 18) was also taken from the rat lactotroph model. However, to preserve the internal Ca^2+^ dynamics under the scaling, the factors *α* (in Eq 18) and *A* (in Eq 15), which converted Ca^2+^ currents into Ca^2+^ concentrations were *multiplied* by *F*_*scale*_. A similar scaling was used in [37].

Second, *I*_*Na*_ and *I*_*Ca*_ were added to the model, with arbitrarily chosen initial conductances.

Third, we tuned the model by adjusting a selection of parameters in order to obtain a firing pattern in closer resemblance with the experimental current-clamp data (Fig 1). The adjustments included: (i) *τ*_*BK*_ was set to 5 ms, a compromise between the time constants of 3 ms and 20 ms used for two different BK channels in the original study [30], and the same value as that used in [32], where a similar model for *I*_*BK*_ was used. (ii) *τ*_*K*_ was set to 5 ms, which was faster than in the rat lactotroph model, where *τ*_*K*_ was based on experimental data where *I*_*K*_ activation had a fast (3.7 ms) and a slow (30 ms) component [63]. The rat lactotroph model used the time constant of the slow component, while we chose a value closer to that of the fast component, as this was due to the faster *I*_*Na*_-mediated APs seen in medaka gonadotroph. (iii) Through manual trial and error, the conductances of all ion channels were tuned freely in order to adapt the medaka gonadotroph model to the experimental data, both in terms of the control conditions and under application of paxilline. The final set of conductances were those given in Table 1.
